# Radiation-Induced Childhood Thyroid Cancer after the Fukushima Daiichi Nuclear Power Plant Accident

**DOI:** 10.3390/ijerph21091162

**Published:** 2024-09-01

**Authors:** Yoshihiro Sokawa

**Affiliations:** Department of Applied Biology, Kyoto Institute of Technology, Kyoto 606-8585, Japan; sokawa@snr.kit.ac.jp

**Keywords:** Fukushima Nuclear Power Plant accident, childhood thyroid cancer, thyroid ultrasound examination, common case, radiation-induced case

## Abstract

After the Fukushima Nuclear Power Plant accident in March 2011, a large-scale ultrasound examination of childhood thyroid cancer for all Fukushima residents aged 18 years old or younger was initiated. Fukushima was divided into four areas according to the external radioactivity released by the accident: the highest (A), high-intermediate (B), low-intermediate (C), and the lowest (D). Five rounds of surveys were carried out from October 2011 to March 2023. The annual incidence rates of the “Common Case” not affected by the accident were able to be estimated. The difference between the incidence rate of whole patients and the “Common Case” is that of the “Radiation-induced Case”. The annual incidence rate of the “Radiation-induced Case” began to increase immediately after the accident, where the highest level was seen in A area, and the order was A > B > C > D. It showed that the development of childhood thyroid cancer was affected by the radiation released by the accident. The effect of the radiation consisted of two phases: the first phase may have been due to the damage to the immune system, and the second phase may have been due to the genetic mutation in the children who were youngest at the time of the accident.

## 1. Introduction

A large number of radiation-induced thyroid cancers among children were identified after the Chernobyl Nuclear Power Plant accident in 1986 [1,2,3]. Following the Fukushima Daiichi Nuclear Power Plant (NPP) accident in March 2011, the Fukushima Prefectural Government and Fukushima Medical University initiated thyroid ultrasound examinations for all Fukushima residents, with about 380,000 aged 18 years old or younger at the time of the accident [4,5]. This is the first time that such a large-scale examination of childhood thyroid glands has been carried out anywhere in the world.

The Basic Survey (BS) started from October 2011 to March 2014 to find patients with thyroid cancer before the accident [6]. In Belarus and Ukraine, the incidence of childhood thyroid cancer began to increase between 4 and 5 years after the Chernobyl accident [1,2,3].

Upon the implementation of examinations, Fukushima Prefecture was divided into four different areas according to the external radioactivity released by the NPP accident [5,6]: the highest level of radioactive contamination area of the 13 municipalities of the evacuation area (A); the high intermediate radioactive levels of 12 municipalities of the Naka-dori area (B); the low intermediate radioactive levels of 14 municipalities of the Naka-dori, together with 3 municipalities of the Hama-dori except areas A and B (C); and the lowest level of 17 municipalities of the Aizu region (D) (Supplemental Figure S1).

The BS found that 116 persons were diagnosed with malignancy or suspected malignancy by fine needle aspiration cytology, with 15 in A area, 56 in B area, 33 in C area, and 12 in D area [6]. These results had a huge impact as the Japanese National Cancer Center reported that the Japanese mean annual incidence rate for thyroid cancer among persons aged 19 years old and younger is 2 per 1,000,000 [7]. The results of the BS posed a question of whether the large number of childhood thyroid cancers observed was induced by the NPP accident [8], or was it a result of the large-scale screening of the cancer [9].

After the BS, a Full-Scale Survey (FSS), which was planned to be carried out every 2 years, began in April 2014 to find persons who had developed thyroid cancer after the NPP accident [10]. The first FSS was carried out in the 2014 and 2015 fiscal years [10], the second FSS was in the 2016 and 2017 fiscal years [11], and the third FSS was in the 2018 and 2019 fiscal years [12]. The fourth FSS was carried out in the 2020, 2021, and 2022 fiscal years, due to the coronavirus pandemic [13].

The aim of this paper is to investigate the cause of the development of childhood thyroid cancer in Fukushima after the NPP accident using the analysis of the results of the BS and four rounds of FSS.

## 2. Materials and Methods

The basic information on the number of objects, examinees, and patients with thyroid cancer was obtained from the reports published by the Fukushima Health Management Survey [6,10,11,12,13].

The subjects, who were determined as having either a nodule > 5 mm or a cyst > 20 mm diameter in their thyroids by ultrasound examination and diagnosed with malignancy or suspected malignancy by fine needle aspiration cytology, underwent follow-up or surgical treatment. Of 116 subjects of the BS, 102 had surgery, and 1 was determined to be benign. Of 186 subjects of FSS, 155 had surgery, and all were malignant. In this paper, the subjects diagnosed with malignancy or suspected malignancy were defined as patients with childhood thyroid cancer.

There were no cases in children under the age of 7 during the 5 rounds of the survey, and no one complained of subjective symptoms before finding thyroid cancer in the surveys. The gender ratio was 1 boy:1.3 girls during the 1st to 3rd FSS. From these features, the data of the BS and FSS were adjusted as follows: the cancer was determined at first by the present surveys; no distinction between girls and boys; the objects and examinees aged 5 years or younger in the BS and those aged 7 years or younger in FSS were eliminated from the data, and upper age of the objects of FSS was 24 years old. In FSS, no patient who developed symptoms before the Fukushima Daiichi NPP accident was present. The data of the BS and four rounds of FSS are shown in Table 1.

## 3. Results and Discussion

### 3.1. Estimation of the Incidence Rate at the Time of Fukushima NPP Accident

At first, we attempted to estimate the incidence rate (number of patients/10^5^ examinees) of childhood thyroid cancer in Fukushima at the time of the NPP accident. Table 2 shows the incidence rate observed in each of four areas of A, B, C, and D in the BS and the first FSS, together with the cumulative sum of both incidence rates. Elapsed times (years) from the accident were shown.

In order to estimate the incidence rate of thyroid cancer at the time of the accident, the incidence rate of the BS and the cumulative sum of the incidence rate of the BS and that of the first FSS were plotted against the time of the surveys, and the cumulative lines were extrapolated to the origin of the coordinate axis of March 2011 when the accident occurred.

**Figure 1:**
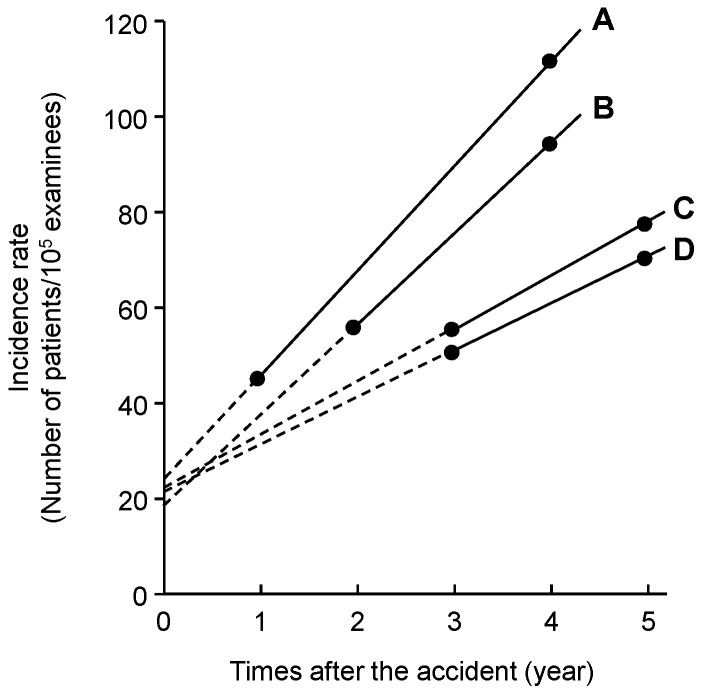
Cumulative lines of A, B, C, and D areas. Data are shown in Table 2.

Figure 1 shows each of the cumulative lines of A, B, C, and D areas. The vertical axis indicates the incidence rate of thyroid cancer. The horizontal axis indicates the elapsed time (years) from the Fukushima NPP accident. The order of the incline of each four lines was A > B > C > D, which clearly indicates that the NPP accident affected the incidence of childhood thyroid cancer in Fukushima.

The intersection point of each extension line with the vertical axis indicates the incidence rate at the time of the accident, which was not affected by the radiation exposure. Each of the four lines intersected at almost the same point; 23.8 for A, 19.3 for B, 22.0 for C, and 22.5 for D. The mean was 21.9 ± 2.7.

### 3.2. Estimation of the Annual Incidence Rate after the Accident in BS and 4 Rounds of FSS

Next, we estimated the annual incidence rate (number of patients/10^5^ examinees/year) after the accident of each of the four areas in the BS and four times of FSS. The annual incidence rate was obtained by dividing the incidence rate by the inspection period (year). The incidence rate in the BS after the accident was obtained by the difference between the incidence rate at each survey (Table 2) and that at the time of the accident, i.e., 21.9. The incidence rate of each FSS was calculated by the data indicated in Table 1. Table 3 shows the annual incidence rate after the accident In the BS and 4 rounds of FSS.

### 3.3. Annual Incidence Rate of “Common Case” before and after the Accident

**Figure 2:**
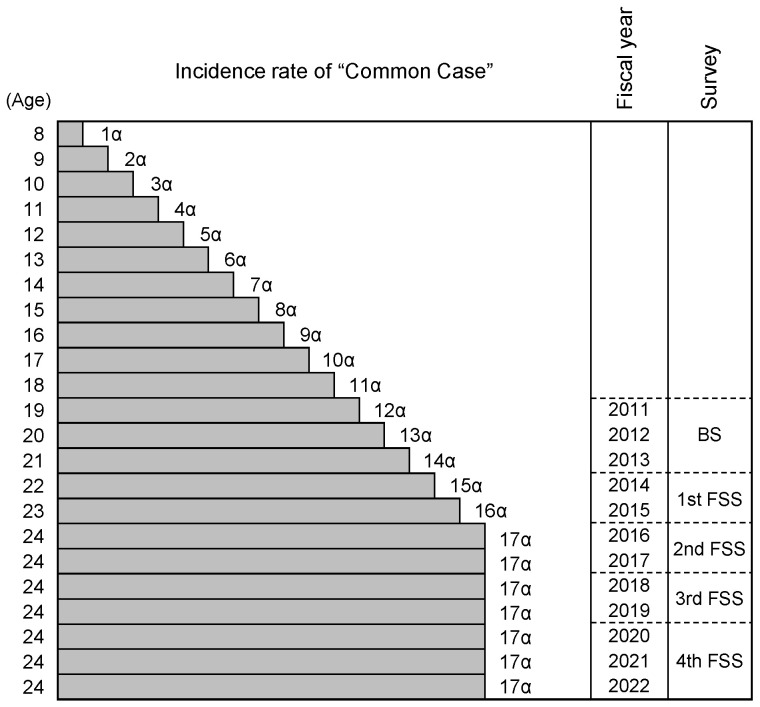
Incidence rate of “Common Case” of each age before and after the Fukushima NPP accident. The symbols on the right side of the bars correspond to the incidence rate of a given age. BS: Basic Survey; FSS: Full-Scale Survey.

As mentioned above, thyroid cancer was not found in children aged 7 years old or younger, and we assumed that the incidence of the “Common Case” not affected by the NPP accident increased at the same rate over 8 years old in both girls and boys. Figure 2 shows the incidence rate of the “Common Case” of each age before and after the accident, where the upper limit of age in FSS was 24 years old, in which the rate was designated by α (number of patients/10^5^ examinees/age).

The total incidence rate of thyroid cancer (from 8 to 18 years old) before the accident was assumed as follows,

1α + 2α + 3α + ……… + 9α + 10α + 11α = 66α

As shown above, the incidence rate of thyroid cancer (number of patients/10^5^ examinees) at the time of the accident was inferred as 21.9, then α can be calculated as 0.332 (= 21.9/66).

Using the above results, I could calculate the annual incidence rate of the “Common Case” at and after the accident in A, B, C, and D areas in the BS and 4 rounds of FSS. The results are shown in Table 4.

### 3.4. Transition of the Annual Incidence Rate of Thyroid Cancer during Surveys after the Fukushima Daiichi NPP Accident

**Figure 3:**
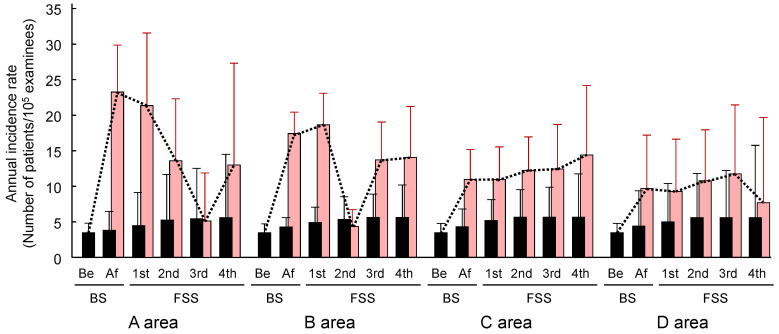
Annual incidence rate after the accident in BS and 4 rounds of FSS. BS: Basic Survey; FSS: Full-Scale Survey; Be: Before accident; Af: After accident. Red bars show the annual incidence rate of whole patients. Black bars show the annual incidence rate of “Common Case”. Upward straight lines on the bars show the half length of a 95% Confidence interval. Data are shown in Table 3 and Table 4.

The results of Table 3 and Table 4 are shown together in Figure 3, which shows the transition of the annual incidence rate of thyroid cancer in each of the four areas during the surveys after the NPP accident. The difference between the annual incidence rate of whole patients and that of the “Common Case” is equivalent to that of the “Radiation-induced Case”. The annual incidence rate of the “Radiation-induced Case” of each of the four areas increased immediately after the accident; the highest rate was seen in A area and the order was A > B > C > D. This shows that the development of childhood thyroid cancer after the accident was affected by the radiation released from the NPP accident.

In A and B areas, the level of the annual incidence rate once rose, then fell to near the 0-time when the accident occurred; however, it rose again to a moderate level. Thus, the effect of radiation released by the Fukushima NPP accident on the development of childhood thyroid cancer consisted of two phases. In C and D areas, this characteristic V-shaped change was not seen, probably due to the low level of the radiation.

Recently, a challenging theory of “cancer immunoediting” has been advocated and is attracting a great deal of attention [14,15]. Tumor cells generated by genetic mutation undergo the cancer immunoediting process before generating clinical cancer. Furthermore, the “cancer-immunity cycle” in which the immune system recognizes and eliminates cancer cells has been proposed [16]. The immune system plays an important role in cancer development, progression, and elimination.

We would like to propose that the immediate increment of childhood thyroid cancer after the NPP accident, the first phase of the radiation-induced incidence, was due to the damage to the immune system by radiation exposure and that the extent of the damage was proportional to the level of radiation exposed.

We have not yet obtained direct evidence of the effect of radiation exposure on the cancer immunoediting process or on the cancer-immunity cycle. However, Ochiai et al. reported low white and red blood cell counts, hemoglobin, and hematocrit in Fukushima monkeys after the Fukushima NPP accident [17]. Urushihara et al. also reported that in Japanese macaque in Fukushima, the white blood cell and platelet counts in peripheral blood, and the myeloid cells and megakaryocytes in bone marrow, showed an inverse correlation with the internal dose rate [18]. After the Chernobyl NPP accident, reduced blood cell counts, hemoglobin, and platelet counts in Ukrainian children have been observed [19].

Regarding the effect of radiation released by the Fukushima NPP accident on the incidence of thyroid cancer, two conflicting opinions have been reported; one is that the radiation affected on a large scale [8,20,21,22], and the other is that the radiation effects have not been observed due to low-dose radiation exposure [9,23,24,25], and serious debate has arisen between them [26,27]. However, the former did not concern itself with the presence of the “Common Case” of childhood thyroid cancer, and the latter could not explain the geographic difference between the four areas in the “Radiation-induced Case”.

Four years after the Chernobyl accident, the Chernobyl Sasakawa Health and Medical Cooperation Project started a mass examination for childhood thyroid cancer using highly sensitive ultrasound equipment, and began to discover many patients in Belarus, Russian Federation, and Ukraine [28]. If mass screening by ultrasound equipment was introduced from the beginning of the examination, many more patients may have been found during the early stages considering the asymptomatic nature of thyroid cancer. There is a possibility that the first phase of the radiation-induced incidence seen in Fukushima was overlooked in Chernobyl.

Following the decrease in the incidence rate observed in areas A and B in the second or third FSS, the rate increased again in the third or fourth FSS. The second phase of the radiation-induced incidence may be the result of the effect on the genetic mutation by radiation exposure of children who were youngest at the time (0–4 years of age). It has been reported that the incidence of childhood thyroid cancer began to increase between 4 and 5 years after the Chernobyl accident, and the increases were particularly pronounced among children youngest at the time of exposure [1,2,3].

Iwadate et al. reported that the BRAF^V600E^ mutation detected mainly in the patients observed in the BS and the first FSS in Fukushima, which was different largely from the Chernobyl case, resembles rather the feature of Japanese adult patients [29]. An investigation of the gene mutation in childhood thyroid cancer after the third FSS (the second phase of the radiation-induced incidence) is expected.

I propose that the radiation released by the Fukushima NPP accident had two different effects on the development of childhood thyroid cancer. An early effect, which may have been due to the damage to the immune system, and a late effect, which may have been due to the genetic mutation in the youngest children at the time; however, we have not obtained direct evidence of these effects.

## Figures and Tables

**Table 1:** Data of Basic Survey (BS) and 4 rounds of Full-Scale Survey (FSS).

Survey	Area	Inspection Period (Year)	No. of Objects	No. of Examinees *	No. of Patients
BS	A	1	35,014	30,605 (87.4%)	14
	B	2	114,825	98,808 (86.1%)	56
	C	3	78,616	59,714 (76.0%)	33
	D	3	36,772	23,552 (64.0%)	12
	Total		265,227	212,679 (80.2%)	115
1st FSS	A	3	37,025	25,873 (69.9%)	17
	B	2	125,731	93,583 (74.4%)	35
	C	2	89,585	62,953 (70.3%)	14
	D	2	42,224	26,271 (62.2%)	5
	Total		294,565	208,680 (70.8%)	71
2nd FSS	A	2	35,154	214,25 (60.9%)	6
	B	2	120,103	78,546 (65.4%)	7
	C	2	86,162	53,468 (62.1%)	13
	D	2	39,315	23,100 (58.8%)	5
	Total		280,734	176,539 (62.9%)	31
3rd FSS	A	2	33,382	19,108 (57.2%)	2
	B	2	115,663	72,346 (62.5%)	20
	C	2	82,233	48,271 (58.7%)	12
	D	2	37,131	21,099 (56.8%)	5
	Total		268,409	160,824 (59.9%)	39
4th FSS	A	3	32,140	14,787 (46.0%)	6
	B	3	112,762	54,391 (48.2%)	23
	C	3	74,592	31,990 (42.9%)	14
	D	3	33,444	12,791 (38.2%)	3
	Total		252,938	113,959 (45.1%)	46

* Parentheses indicate the rate of examination (%).

**Table 2:** The cumulative sum of the incidence rate of BS and 1st FSS.

Area	BS		1st FSS		BS and 1st FSS
	Elapsed Time (year) *	Incidence Rate **	Elapsed Time (Year) *	Incidence Rate **	Cumulative Sum
A	1	45.7	4	65.7	111.4
B	2	56.7	4	37.4	94.1
C	3	55.3	5	22.2	77.5
D	3	51.0	5	19.0	70.0

* Elapsed time shows the duration between the time of the accident and the end of the survey. ** No. of patients/10^5^ examinees.

**Table 3:** Annual incidence rate after the accident in BS and 4 rounds of FSS.

Survey	Area	Incidence Rate *	Inspection Period (Year)	Annual Incidence Rate ** (95% CI ***)
BS	A	45.7 − 21.9 = 23.8	1	23.8 (17.7, 29.9)
	B	56.7 − 21.9 = 34.8	2	17.4 (14.4, 20.5)
	C	55.3 − 21.9 = 33.4	3	11.1 (7.0, 15.2)
	D	51.0 − 21.9 = 29.1	3	9.7 (2.2, 17.3)
1st FSS	A	65.7	3	21.9 (11.9, 31.7)
	B	37.4	2	18.7 (14.3, 23.1)
	C	22.2	2	11.1 (6.6, 15.6)
	D	19.0	2	9.5 (2.3, 16.7)
2nd FSS	A	28.0	2	14.0 (5.7, 22.3)
	B	8.9	2	4.5 (2.2, 6.8)
	C	24.3	2	12.2 (7.4, 17.0)
	D	21.6	2	10.8 (3.6, 18.0)
3rd FSS	A	10.5	2	5.2 (−1.5, 11.9)
	B	27.6	2	13.8 (8.6, 19.1)
	C	24.9	2	12.4 (6.1, 18.8)
	D	23.7	2	11.8 (2.2, 21.5)
4th FSS	A	40.6	3	13.5 (2.0, 27.3)
	B	42.3	3	14.1 (6.9, 21.3)
	C	43.8	3	14.6 (4.6, 24.6)
	D	23.5	3	7.8 (−4.2, 19.8)

* No. of patients/10^5^ examinees. ** No. of patients/10^5^ examinees/year. *** 95% Confidence Interval (CI).

**Table 4:** Annual incidence rate of “Common Case” at and after the accident.

Survey	Area	Incidence Rate *	Inspection Period (Year)	Annual Incidence Rate ** (95% CI ***)
BS at the time of accident	all areas	11α **** = 3.65	-	3.65 (2.47, 4.83)
BS after the accident	A	12α = 3.98	1	3.96 (1.47, 6,45)
	B	(12 + 13)α = 8.30	2	4.15 (2.66, 5.64)
	C	(12 + 13 + 14)α = 12.95	3	4.32 (1.75, 6.89)
	D	(12 + 13 + 14)α = 12.95	3	4.32 (−0.71, 9,35)
1st FSS	A	(13 + 14 + 15)α = 13.94	3	4.65 (0.10, 9.20)
	B	(14 + 15)α = 9.63	2	4.82 (2,59, 7.05)
	C	(15 + 16)α = 10.29	2	5.15 (2.11, 8.19)
	D	(15 + 16)α = 10.29	2	5.15 (−0.18, 10.48)
2nd FSS	A	(16 + 17)α = 10.96	2	5.48 (−0.70, 11.66)
	B	(16 + 17)α = 10.96	2	5.48 (2.44, 8.52)
	C	(17 + 17)α = 11.29	2	5.64 (1,72, 9.56)
	D	(17 + 17)α = 11.29	2	5.64 (−0.57, 11.85)
3rd FSS	A	(17 + 17)α = 11.29	2	5.64 (−1.32, 12.60)
	B	(17 + 17)α = 11.29	2	5.64 (2.29, 8.99)
	C	(17 + 17)α = 11.29	2	5.64 (1.33, 9.95)
	D	(17 + 17)α = 11.29	2	5.64 (−1.00, 12.28)
4th FSS	A	(17 + 17 + 17)α = 16.93	3	5.64 (−3.25, 14.53)
	B	(17 + 17 + 17)α = 16.93	3	5.64 (1.11, 10.17)
	C	(17 + 17 + 17)α = 16.93	3	5.64 (−0.57, 11.85)
	D	(17 + 17 + 17)α = 16.93	3	5.64 (−4.57, 15.85)

* No. of patients/10^5^ examinees. ** No. of patients/10^5^ examinees/year. *** 95% Confidence interval (CI). **** α = 0.332.

## Data Availability

Publicly available data reported by Fukushima Health Management Survey were analyzed in this study. These data can be found in https://www.pref.fukushima.lg.jp/uploaded/attachment/461398.pdf; https://www.pref.fukushima.lg.jp/uploaded/attachment/238768.pdf; https://www.pref.fukushima.lg.jp/uploaded/attachment/401325.pdf; https://www.pref.fukushima.lg.jp/uploaded/attachment/529187.pdf; https://www.pref.fukushima.lg.jp/uploaded/attachment/644647.pdf (in Japanese).

## Supplementary Materials

The following supporting information can be downloaded at: https://www.mdpi.com/article/10.3390/ijerph21091162/s1, Figure S1: Map of 4 areas in the Fukushima Health Management Survey (FHMS)—-division of Fukushima prefecture.

## Abbreviations

The following abbreviations are used in this manuscript.

NPP	Nuclear Power Plant
BS	Basic Survey
FSS	Full-Scale Survey
95% CI	95% Confidence interval
