# Supplementary material for: Radiation-Induced Childhood Thyroid Cancer after the Fukushima Daiichi Nuclear Power Plant Accident
Source: Int J Environ Res Public Health. 2024 Sep 1;21(9):1162. doi: 10.3390/ijerph21091162 (PMC11431259; doi:10.3390/ijerph21091162)
Supplement: Supplementary file 1 [file ijerph-21-01162-s001.zip › ijerph-3167720-supplementary.pdf]

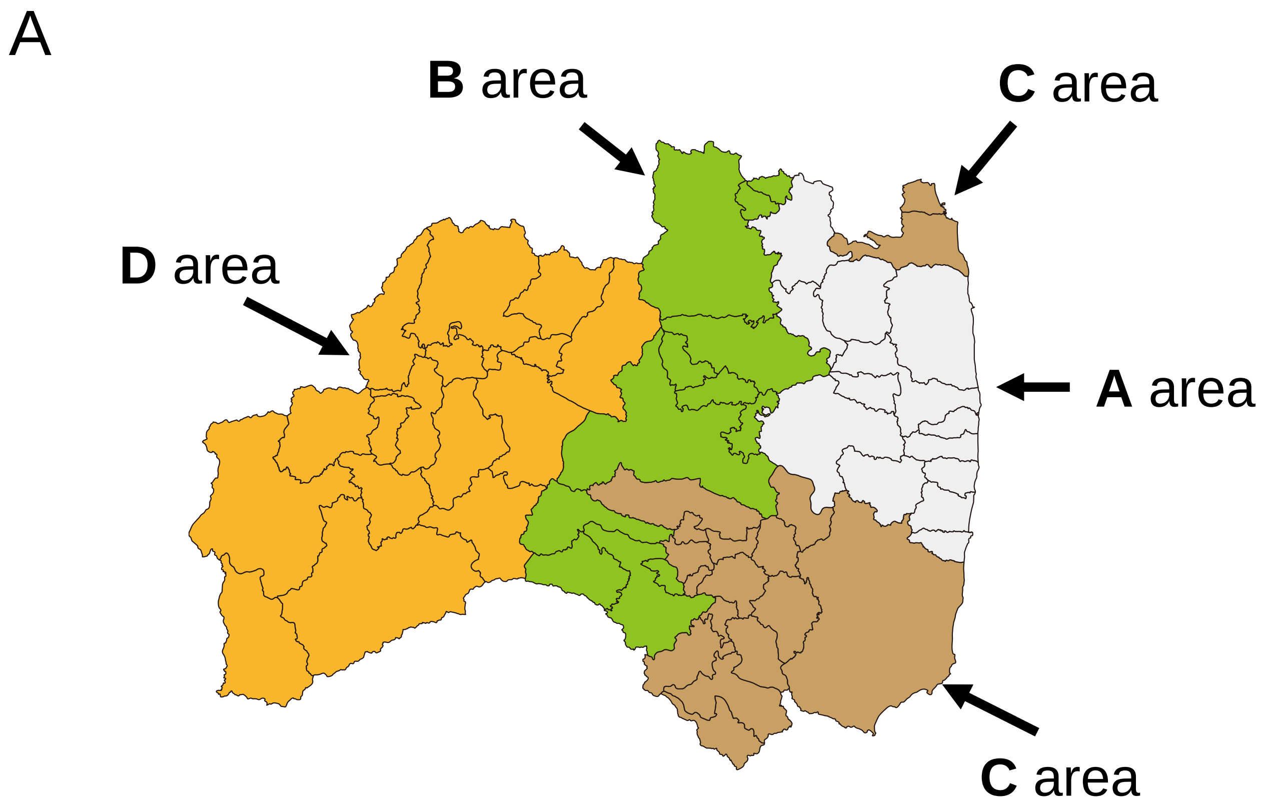

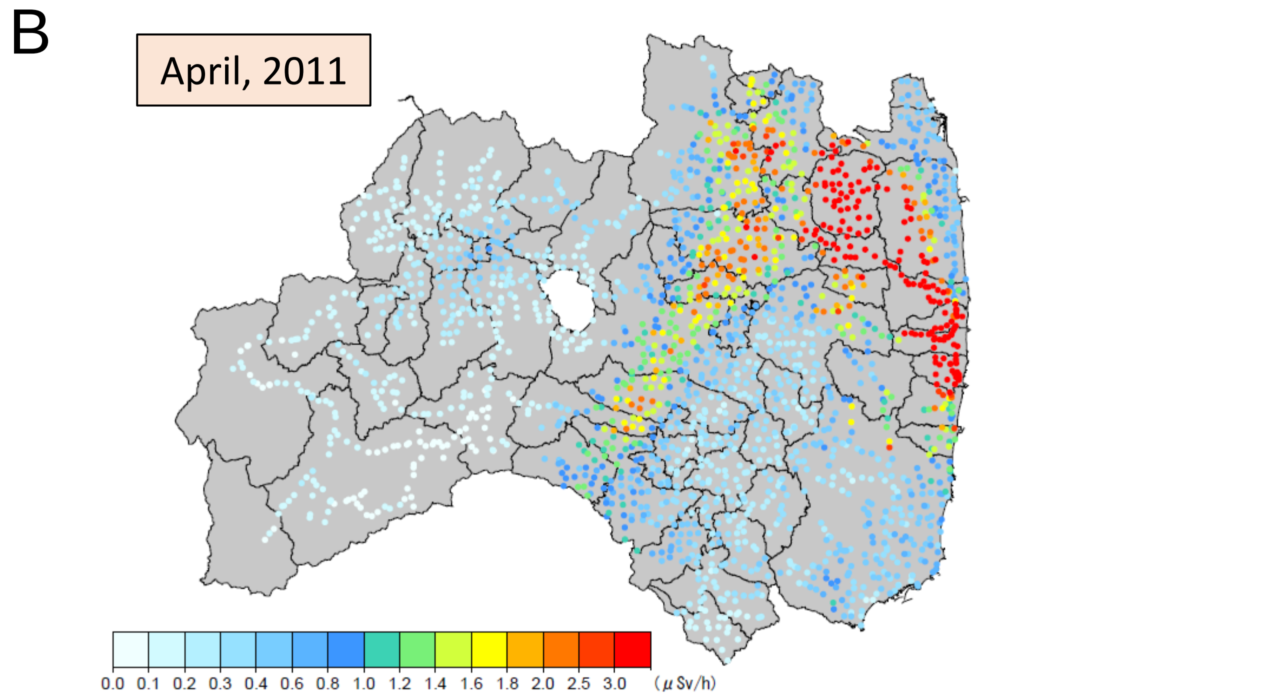

Supplemental Figure S1. Map of 4 areas in the Fukushima health management survey (FHMS)-division of Fukushima prefecture

A. Map of 4 areas in the Fukushima Prefecture; **A** area (highest), **B** area (high intermediate), **C** area (low intermediate), and **D** area (lowest).

B. Radiation dose rates at the time of the accident (Fukushima prefecture radioactivity measurement map; https://fukushima-radioactivity.jp/pc/)
